# Annual Research Review: How did COVID‐19 affect young children's language environment and language development? A scoping review

**DOI:** 10.1111/jcpp.14102

**Published:** 2024-12-27

**Authors:** Cecilia Zuniga‐Montanez, Catherine Davies, Laurie Ligoxygakis, Draško Kašćelan, Nayeli Gonzalez‐Gomez

**Affiliations:** ^1^ School of Languages, Cultures and Societies University of Leeds Leeds UK; ^2^ Centre for Psychological Research Oxford Brookes University Oxford UK; ^3^ School of Health and Social Care University of Essex Colchester UK

**Keywords:** Scoping review, COVID‐19, language development, literacy, early years, primary education

## Abstract

A diverse body of research conducted since the start of Covid‐19 has investigated the impact of the pandemic on children's environments and their language development. This scoping review synthesises the peer‐reviewed research literature on this topic between 2020 and 2023. Following the Joanna Briggs Institute methodology and the PRISMA extension for scoping reviews, we searched five databases for studies that fulfilled the following inclusion criteria: studies with neurotypical (monolingual or multilingual) 0‐6‐year‐old children; studies focusing on any area of language development, including sources describing literacy or educational practices that impacted language development; studies focusing in the context of the COVID‐19 pandemic, with no restrictions of geographical location or language used by participants. Ninety‐four eligible studies were identified for review. The extracted data were synthesised using frequency tables and narrative descriptions. Eligible studies used a wide range of data collection periods, methods, research sites, sample ages, sizes, and roles to fulfil 15 broad aims. They show that children's language‐learning environments were significantly impacted, with variability over time and across the socioeconomic spectrum. Together they investigated diverse language domains, as well as several home, educational, and demographic factors that were hypothesised to impact children's language development. Of those studies that focused on language outcomes, most converge to suggest a decline in typical expectations of children's language development, including their social communication, vocabulary, morphosyntax, literacy, and language of schooling, as well as general communication skills, school readiness, and other areas of academic progress. Our synthesis suggests that children's language and environment were significantly impacted by COVID‐19. This scoping review will support families, researchers, practitioners, and policymakers working with pandemic‐era children to further understand the effects of the pandemic on children's development.

## Introduction

A child's early communication environment is a critical determinant of their language development (Gilkerson et al., 2018; Roulstone, Law, Rush, Clegg, & Peters, 2011), which in turn impacts their later educational, social, and economic outcomes (Blanden, 2006; Downer & Pianta, 2006; Melhuish et al., 2008; Rodriguez et al., 2009). A broad evidence base shows the influence of a range of environmental factors on language. These include: (a) *home language and literacy environment*, e.g., quantity and quality of child‐directed speech, shared book‐reading, use of technology for academic purposes (Hirsh‐Pasek et al., 2015; Huttenlocher, Haight, Bryk, Seltzer, & Lyons, 1991; Melhuish et al., 2008; Miser & Hupp, 2012; Noble et al., 2019; Romeo et al., 2018; Rowe, 2012; Schwab & Lew‐Williams, 2016; Weisleder & Fernald, 2013; Weizman & Snow, 2001); (b) *daily activities*, e.g., caregiver‐child activities (Dore, Logan, Lin, Purtell, & Justice, 2020; Karani, Sher, & Mophosho, 2022; Kartushina et al., 2022; Operto et al., 2020), digital media exposure; (c) *parenting behaviours*, e.g., caregiver sensitivity and attitudes, maternal mental health (Hurtado, GrÜTer, Marchman, & Fernald, 2014; McGillion, Davies, Kong, Hendry, & Gonzalez‐Gomez, 2023; Noble et al., 2015; Rowe, Pan, & Ayoub, 2005; Tamis‐LeMonda, Bornstein, & Baumwell, 2001) (d) *educational factors*, e.g., attendance at early childhood education and care (Davies et al., 2021, 2023; Geoffroy et al., 2007; Melhuish & Gardiner, 2018, 2020; Sylva, Melhuish, Sammons, Siraj‐Blatchford, & Taggart, 2004) and (e) *family demographics*, e.g., parental educational level and other indices of socioeconomic status (Bornstein, Haynes, & Painter, 1998; Hoff, 2006; Hoff‐Ginsberg, 1998). Overall, these and other environmental factors have been found to influence a range of measures of child language, including utterance length and complexity, narrative skills, print awareness, phonological awareness, vocabulary breadth and depth, processing speed, and neural language processing. Taken together, this representative sample of research provides strong evidence that the early communication environment is critical for language development.

The COVID‐19 pandemic and its associated lockdowns had a pervasive effect on children's language environments and exacerbated some of the existing inequalities in language learning opportunities. Social restrictions affected daily activities, e.g., decreased visits to playgrounds and libraries, increased screen time (Bergmann et al., 2022; Chambonniere et al., 2021; Hendry et al., 2022; Kartushina et al., 2022; Schmidt et al., 2020), and curtailed access to education (Davies et al., 2021, 2023; Department for Education, 2021). Changes to employment and increased family stress impacted parenting behaviours as caregivers split their resources between caring for young children, home‐schooling, and working, alongside increased health and economic worries (Calvano et al., 2022; Gadermann et al., 2021). Overlaying these effects, the pandemic had a heavier impact on socioeconomically disadvantaged families. They missed more formal early learning than their more advantaged peers (La Valle et al., 2022) and suffered disproportionately regarding access to services, loss of social support, and increased family stress, illness, and bereavement (Shum et al., 2020).

As multiple environmental aspects are known to impact language development in non‐pandemic contexts, it is important to understand this relationship when predictors change so pervasively. A dense body of research has been generated investigating the impacts of the COVID‐19 pandemic and disruptions on children's environments and their language development. The research to date is diverse: it investigates a wide range of environmental factors and domains of language and literacy, as well as a broad spread of populations, developmental stages, research sites, and national contexts. It analyses qualitative and quantitative data gleaned in many ways, including direct measures, parent/practitioner reports and observations, and interventions. It has been published through peer review and more rapidly through practitioner‐based reports.

Research continues to emerge as families are followed up post‐pandemic. Despite (or perhaps because of) this rapid proliferation of research between 2020 and the time of writing, the extent and nature of pandemic‐related influences on young children's language environments and language development have not yet been scoped. This scoping review provides a much‐needed synthesis of the peer‐reviewed research literature published to date. Following Munn et al. (2018), it systematically scopes the volume, foci, and boundaries of the topic. It surveys (though does not evaluate) the research methods used, as due to pandemic‐related limitations on established methods such as in‐lab testing or school‐based interventions, researchers had to pivot quickly to newer methods such as remote testing and rely on parent‐report and convenience sampling.

A scoping methodology is the ideal tool to collate relevant environmental factors introduced by the pandemic, highlight their likely impacts on children's language development, and identify salient gaps in current knowledge. Our review examines known influences on language development (which may have been exacerbated during the pandemic) as well as how new environmental factors – such as mask‐wearing and degree of pandemic disruption – impacted children's language development. As the pandemic has not impacted everyone equally, these factors will include demographic mediators.

Now 4 years on from the first of multiple lockdowns around the world, as many pandemic‐era babies have entered formal schooling, families, practitioners, and policymakers are concerned about mounting evidence that lockdowns led to delays in key developmental skills, especially in children from socioeconomically disadvantaged backgrounds. Scoping the literature for converging data is an essential step in understanding this evidence base. Our review will be key in devising specific questions on the mechanisms of the pandemic's influence on children's language development. Together with future systematic reviews, this review will ultimately inform: (a) recovery practice such as differentiated school provision, (b) policy to mitigate longer term impacts of COVID‐19 on children as they grow, and (c) responses to future pandemics or comparable events that transform children's learning environments.

To our knowledge, only two related reviews of pandemic effects on language development have been published: a literature review on the impact of social isolation on speech development (Lukić et al., 2022) and a scoping review comparing the efficacy of remote and face‐to‐face speech therapy during the pandemic (Hassanati et al., 2023). The current review takes a more comprehensive approach, scoping literature on a broader range of environmental factors across a fuller range of linguistic domains (e.g., vocabulary, family literacy practices, multilingual exposure). We will focus on children from birth to the point at which they enter formal schooling around age six – a critical window for language development.

### Objectives

To provide a comprehensive picture of the impact of COVID‐19‐related environmental changes on language development, this scoping review identifies and presents the available information published since 2020 regarding factors relating to the home, educational, and demographic environments on a range of 0‐6‐year‐olds' language skills. It aims:

1. To summarise the demographic and methodological characteristics of the evidence base about the impact of COVID‐19 on language development.
2. To identify COVID‐19‐related factors that affected children's *environment*, e.g.,
  - *factors relating to the home (learning) environment*, e.g., social support, family literacy practices, interaction quality/caregiver sensitivity, screen‐ and reading times, mask‐wearing, and caregiver‐child activities.
  - *factors relating to the educational environment*, e.g., access to early years education and care, degree of school disruption, and child engagement in remote learning.
  - *factors relating to caregivers*, e.g., parenting attitudes and parental mental health.
3. To identify the factors or areas of language development investigated during the Covid‐19 pandemic, e.g., vocabulary, narrative, print awareness, print motivation, letter knowledge, phonological awareness, and read‐aloud behaviours.
4. To identify *other variables* that are investigated alongside language outcomes during COVID‐19, e.g., cognitive and physical factors.
5. To identify *demographic variables* moderating points 2 and 3, e.g., how socioeconomic background, neighbourhood deprivation, child age, the number of children in the family, parents' educational level, and multilingual status might affect the strength of factors identified above.
6. To consider the *likely effects* of the factors and variables identified in 2–4.

At the end of the scoping review, we reflect on its findings to make recommendations for researchers, families, practitioners, and policymakers supporting children as they move through education and planning for mitigations during comparable future events.

## Methods

**Figure 1:**
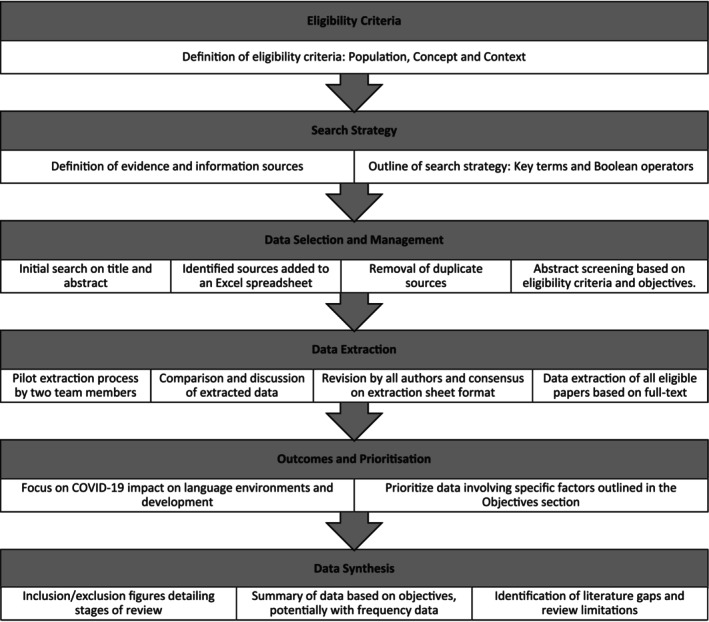
Summary of scoping review process

This scoping review was conducted following the Joanna Briggs Institute (JBI) methodology for scoping reviews (Peters et al., 2020). We were guided by Arksey and O'Malley (2005), Munn et al. (2022), and Tricco et al. (2018). The PRISMA extension for scoping reviews (Tricco et al., 2018) was used for reporting our findings. Figure 1 shows a summary of the scoping review process.

### Protocol and registration

A protocol for the current scoping review was drafted (guided by Peters et al., 2022) and can be found on our project page at https://osf.io/4u8dw/. Due to time constraints, the drafting of the protocol took place in parallel with the initial literature search and data extraction but prior to any analysis. Nevertheless, the protocol outlines the originally planned aims of the scoping review, which the review team settled on before the initial literature search. Any discrepancies from the protocol that might have emerged based on the analysis stage are acknowledged in this paper.

### Eligibility criteria

Studies published between 2020 (the earliest date that our context keywords are mentioned) and 2023 (when the database search was conducted) that met the eligibility criteria below were included in this scoping review. No restrictions were imposed on the methods used in the studies included in this scoping review. Studies reported in English, Spanish, or Serbo‐Croatian (Bosnian/Croatian/Montenegrin/Serbian [BCMS]) were included as these were languages spoken/understood by members of the reviewer team. Sources which had the full text available were included. We also included those with only the title and abstract available (e.g. due to embargo), if these suggested that the paper was relevant to the scoping review.

#### Population

Studies had to focus on children from birth to 6 years of age. Studies that additionally involved older children were also included as long as the sample included children within our target age range. This was decided because a significant number of studies that include children up to 6 years of age might also include some older children. Studies that directly assessed children were included, but also studies that reported the experiences and perspectives of parents, caregivers, educators, or other adults regarding children's language development. No restrictions were set on the number of languages spoken or understood by participants. Studies were excluded if the authors reported that any of the participants had a condition or disorder that could impact language development. By excluding studies involving children with conditions or disorders known to impact language development, the review can more accurately describe the pandemic's effects on typical language development, avoiding confounding variables introduced by pre‐existing conditions or disorders.

#### Concept

Studies focusing on any area of language development were considered, including literacy or educational practices that impacted children's language development. Studies that assessed the role of children's environments and parental characteristics on language development during the pandemic were also included.

#### Context

Studies conducted in the context of the COVID‐19 pandemic and how it impacted children's language development were included. No restrictions were set on the particular language used by study participants or the study's geographical location.

### Information sources and search strategy

To identify relevant research, an initial literature search was conducted between August and October 2023 in the following databases: Web of Science, OVID, PubMed, PsychInfo, and ProQuest. The key terms and search strategies were defined by the reviewer team in conjunction with a research librarian. The key terms used in the initial literature search can be found in Table 1.

**Table 1:** Key terms used in the Initial Literature Search

Key terms 1 (population)	Key terms 2 (concept)	Key terms 3 (context)
child*	language	covid*
infant*	language development	covid 19
bab*	language acquisition	pandemic
toddler*	communication	post‐pandemic
early years	understand*	post pandemic
early childhood	comprehension	
kindergarten	produc*	
nurser*	expressive vocabulary	
preschool*	vocabulary	
reception	semantics	
KS1/Key Stage 1	phonology	
bilingual*	syntax	
multilingual*	pragmatics	
caregiver*	literacy	
parent*	reading	
	writing	
	speech	
	talk*	
	word*	

The initial literature search was conducted by one member of the reviewer team. To be eligible for this scoping review, studies had to include at least one key term from each category in this initial search in the title and/or abstract. All three categories of the key terms were either searched together or using the databases' combined search function (see example below). The search strategy used on each database, as well as any filters and limits used, can be found in Table S1 and at https://osf.io/4u8dw/.

#### Example search

(Child* OR infant* OR bab* OR toddler* OR “early years” OR “early childhood” OR kindergarten OR nurser* OR preschool* OR reception OR KS1 OR “Key Stage 1” OR bilingual* OR multilingual* OR caregiver* OR parent*) AND (language OR “language development” OR “language acquisition” OR communication OR understand* OR comprehension OR produc* OR “expressive vocabulary” OR vocabulary OR semantics OR phonology OR syntax OR pragmatics OR literacy OR reading OR writing OR speech OR talk* OR word*) AND (covid* OR “covid 19” or pandemic OR post‐pandemic OR “post pandemic”).

Primary research studies were included. If a meta‐analysis or a systematic review was identified, the original papers that the reviews synthesised were screened and included if they fulfilled our inclusion criteria. The meta‐analyses or systematic reviews were then removed from our corpus to avoid data duplication. Only one systematic review was identified in the initial literature review (Betthäuser, Bach‐Mortensen, & Engzell, 2023). Grey literature (i.e., preprints, practitioner reports, and policy documents) were included if they were discussed in an included systematic review and if they were relevant to the scoping review. A separate search of the grey literature was not conducted due to time and resource limitations.

To select the sources of evidence, one member of the reviewer team screened the title and abstract of all studies identified in the initial literature search. If this reviewer was unsure about the eligibility of any of the studies, a second reviewer screened those studies. The full text of all the studies that met the inclusion criteria was then reviewed by two reviewers, and any studies that did not fulfil the criteria were excluded. Any inconsistencies or disagreements were resolved in consultation with a third reviewer.

### Data items and data extraction process

To identify the data needed for this scoping review, a data extraction sheet was created by two members of the reviewing team. They first conducted a pilot of the data extraction process by randomly selecting the same three studies from the identified corpus and then independently extracted data into a data extraction sheet, which they had jointly developed. The same two reviewers then randomly and independently selected three more studies and extracted the data. Both then compared extractions and agreed on the proposed format of the data extraction sheet. The whole reviewing team then reviewed and discussed the extraction sheet/extraction process and agreed on the final data extraction sheet/process. The data extraction sheet (template and completed version) can be found at our project page at https://osf.io/4u8dw/.

To extract the relevant data, one of the two reviewers who created the data extraction sheet read all eligible sources of evidence (if available) and extracted the data. A second member of the reviewer team reviewed the extracted data to ensure that it was entered consistently and that no information was missing. Note that two texts were revised by only one member of the reviewing team because they were written in Spanish or Croatian. Our data items related to:

- characteristics of the paper, i.e., full reference, the affiliation of the first author, the type of text (e.g., journal article, dissertation, etc.), and the study site(s)
- sample characteristics, i.e., the language used to assess children, sample age, size, and role (e.g., children, parents, educators, practitioners) and further details of their characteristics (e.g., gender, languages used/spoken)
- study aims
- method (i.e., data collection period, method type, method characteristics, assessments or measurements used), factors investigated or included (e.g., language factors, cognitive factors, home environment, educational practices, or socioeconomic factors), and if language was the main factor assessed
- analytical methods
- main findings
- whether the research conducted was directly linked or related to another paper in our corpus or part of a bigger research project
- further comments or concerns, e.g., regarding the quality of the study
- decisions on study inclusion/exclusion, with reasons.

NB. Auxiliary columns were added during coding/synthesising to facilitate the process, e.g., ‘categorical sample age’, which were simplifications of the data items listed above.

## Results

A total of 8,200 texts were identified after the initial literature search described above. After removing duplicates, one member of the reviewer team screened the title and abstract of all studies. Then, the full text of all the articles that met the inclusion criteria (*N* = 124) was reviewed by two reviewers, and any studies that did not fulfil the criteria were excluded. Three studies were reviewed by a third member of the reviewing team, as the first two were unsure whether they should be included or not. All three texts were included.

**Figure 2:**
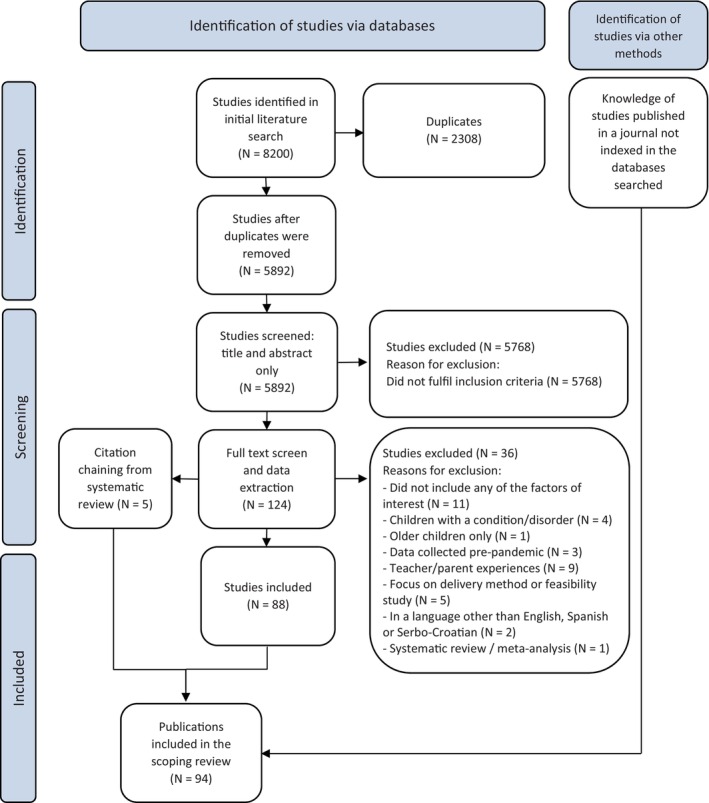
PRISMA flow diagram for selecting studies

After the extraction process, a team member noticed that a relevant article, already familiar to the team, was missing from the database searches (likely due to an indexing issue). Consequently, this article was subsequently incorporated into the findings. A final total of 94 studies went forward for review (see Appendix S1). Figure 2 shows the process of study selection. The number of excluded studies at each stage of the process and the reasons for exclusion are reported in Figure 2. All extracted data from the included studies, along with our search strategy, and a list of studies included in the scoping review can be found at our project page at https://osf.io/4u8dw/.

Synthesised results are reported using frequency tables and narrative descriptions, relating to the objectives of the review. To report the characteristics of each study, results for each column in the data extraction sheet were synthesised using the pivot table function in Excel. This calculated the frequency of all unique categories within each data item. In cases where values fell into two different categories, such as having both France and Japan as research sites, a value was assigned to each category (+1 for France and +1 for Japan). Consequently, in some categories, the total count exceeds the overall number of studies reviewed (*N* = 94). This approach was consistently maintained throughout the synthesis process. The aims of the studies were grouped thematically into 15 categories, and frequencies are reported. For data items including sample characteristics, methods, and factors investigated, data were also coded and grouped into new categories, and their frequencies are reported in a synthesis table. This step was taken to categorise the data for ease of scoping while comprehensively capturing the wide range of values. The main findings of the synthesis are summarised narratively, with implications following in the discussion.

### Objective 1. Demographic and methodological characteristics of the evidence base

Table 2 summarises the characteristics of the evidence base on the impact of COVID‐19 on language development.

**Table 2:** Demographic and methodological characteristics of the reviewed studies

Evidence format	*N*	Research site (continent)	*N*	Language used to assess children	*N*	First author affiliation (continent)	*N*
Chapter	1	North America	43	Arabic	1	North America	42
Dissertation	7	Europe	41	Basque	1	Europe	35
Journal article	82	Asia	21	Catalan	1	Asia	14
Preprint	1	South America	1	Cantonese	1	Africa	1
Research report	3	Africa	1	Dutch	3	South America	1
**Data collection period**		Australia	1	English	48	Australia	1
Pandemic	28	*Broken down by country*		French	2	*Broken down by country*	
Pre‐pandemic + Pandemic	13	Australia	1	Japanese	1	Australia	1
Pre‐pandemic + Pandemic + Post‐pandemic	10	Brazil	1	German	3	Brazil	1
Pandemic + Post‐pandemic	21	Canada	6	Hebrew	1	Canada	6
Pre‐pandemic + Post‐pandemic	4	China	2	Hungarian	1	China	2
Post‐pandemic	6	Croatia	2	Indonesian	1	Croatia	2
Not specified	12	France	2	Italian	2	Cyprus	1
**Method type**		Germany	4	Korean	1	France	1
Mixed	24	Hungary	1	Mandarin	7	Germany	3
Qualitative	9	Indonesia	2	NA	24	Hungary	1
Quantitative	61	Ireland	2	Nepali	1	Indonesia	2
**Method characteristics**		Israel	2	Norwegian	2	Ireland	2
(Auto)/ethnographic approach	2	Italy	2	Polish	1	Israel	1
Government assessment data	5	Japan	1	Portuguese	3	Italy	2
In‐person assessment	18	Nepal	1	Romani	1	Korea	1
In‐person experiment	3	Netherlands	3	Russian	1	Netherlands	2
In‐person intervention	3	Norway	3	Serbian	1	Norway	3
In‐person observations	1	Portugal	2	Spanish	6	Poland	1
Interviews	8	Russia	2	Swedish	1	Portugal	2
Medical records	1	Serbia	1	Thai	1	Serbia	1
Online assessment	10	Singapore	3	Turkish	3	Singapore	3
Online intervention	7	South Africa	1	Multiple languages	12	South Africa	1
Online questionnaire	42	South Korea	1	**Sample age**		Spain	6
Phenomenological design	1	Spain	7	Adults	22	Sweden	1
Questionnaire	8	Sweden	1	1y or under	5	Thailand	2
Video interactions	1	Thailand	2	2y or under	3	Turkey	3
**Language as main factor assessed**		Turkey	4	3y or under	11	UK	7
Yes	46	Turkish Republic of North Cyprus	1	4y or under	3	USA	36
No	48	UK	9	5y or under	8	**Analysis method**	
		USA	37	6y or under	5	ANCOVA	4
		Multiple countries	3	7y or under	8	ANOVA	16
				8y or under	7	Correlations	16
				9y or under	4	Chi‐squared tests	11
				10y or under	5	Content analysis	9
				11y or under	3	Ethnographic analysis	2
				18y or under	4	Factor analyses	2
				Not specified	6	Growth models	2
				**Sample size**		Kruskal–Wallis Tests	2
				<10	3	Latent class clusters analysis	2
				10–50	19	Linear effects models	8
				51–100	15	Regression analyses	32
				101–200	14	Mann–Whitney U test	7
				201–500	10	MANOVA	3
				501–1,000	9	Pair‐wise Games‐Howell tests	1
				1,001–5,000	12	Shapiro–Wilk test	2
				>5,000	12	Structural equation modelling	3
				**Sample role**		T‐test	15
				Caregivers	41	Thematic analysis	6
				Children	64	Wilcoxon signed rank tests	5
				Educators	11		

As summarised in Table 2, the majority of studies in our corpus were journal articles (*N* = 82% or 87%), with a smaller number of dissertations and other forms of evidence. Based on WHO dates for the pandemic duration (March 2020 to May 2021), 11% of all studies collected data spanning pre−/during−/post‐pandemic periods, with 29% focusing on the pandemic period only. Quantitative studies were in the majority (65%), rising to 91% using mixed methods and just 10% using qualitative data. Almost half (45%) of the whole corpus used online questionnaires to collect data. Twenty‐seven percent used in‐person methods and 52% used online methods. Forty‐nine percent of studies used language as the main developmental aspect assessed; those that used an additional aspect of development (e.g., maths or motor skills) accounted for the majority of the studies reviewed (51%). North America and Europe accounted for 90% of the research sites, and Asia for 22%. Only 3% of studies analysed data from multiple countries (Crimon et al., 2022; Kartushina et al., 2022; Stucke, Stoet, & Doebel, 2022). English was used to assess children in 51% of the studies. Of the child‐focused studies (*N* = 66), 37% focused on children aged six or under; the remainder involved additional older children. Over half of the studies used data from adults, e.g., caregivers or educators. The most common sample size was 10–50 participants (20%), though 35% of studies worked with over 500 participants and 25% with over 1,000. Studies by lead authors affiliated with North American institutions dominated (45%), with most of the remainder from Europe (37%) or Asia (15%). Research based in Africa, Australia, and South America each accounted for just 1% of the reviewed studies.

We scoped the research aims of the studies reviewed, as relevant to our keyword lists. The corpus contains a wide range of aims. Table 3 shows these coded into 15 categories, with frequency data.

**Table 3:** Categorised aims of reviewed studies

Aims	*N*
To determine language and/or literacy development	24
To investigate home literacy practices	18
To investigate multilingual experiences and abilities	15
To investigate home learning / home schooling practices and experiences	11
To determine general development (e.g. physical; communicative)	10
To determine the efficacy of a language intervention	10
To determine academic skill development (e.g. school readiness, numeracy, reading, reasoning)	9
To determine the effects of adults' mask‐wearing	7
To determine children's use of digital media	5
To investigate the role of caregiver interaction on language development	5
To investigate family experiences and attitudes to the lockdowns	4
To investigate practitioner experiences and attitudes to the lockdowns	4
To investigate socioeconomic effects on general development (e.g. physical; communicative)	3
To investigate the role of in‐person attendance at an educational setting	3
To investigate practices used in educational settings	1

Unsurprisingly, the most common aim was to determine language and/or literacy development, appearing in 26% of sources, increasing to 35% when coupled with sources looking at wider academic skill development. Investigating the home environment was a common aim, with home literacy environment featuring in 19% of sources, rising to 31% when grouped with sources focusing on home‐learning/home‐schooling practices and experiences. Sixteen percent of the studies focused on bilingual/multilingual experiences and abilities. Eleven percent of studies in the corpus analysed a language intervention. Reflecting the relative lack of purely qualitative work in our review, 8% of studies focused on family or practitioner experiences and attitudes to the lockdowns.

### Objectives 2–5

Objective 2 was to identify factors related to COVID‐19 that affected children's home and educational environments. Objective 3 was to identify the factors or areas of language development investigated during the Covid‐19 pandemic. Objective 4 was to identify other variables investigated in the corpus. Findings addressing objectives 2–4 are presented in Table 4. Objective 5 (to identify demographic variables moderating the factors presented) is included in environmental factors in Table 4.

**Table 4:** Environmental, language, and other factors analysed in the reviewed studies

Environment	*N*	Language development	*N*
*Home environment factors*		*Area of language assessed*	
Activities at home	5	Language and communication development	14
Home digital practices	9	Language comprehension and production	11
Home language environment	4	Literacy	46
Home learning environment	8	Language processing	3
Home literacy practices	12	Vocabulary	28
Parental mental health	3	Multilingual exposure/proficiency	10
Parental practices	5	Grammar/syntax/morphology	11
Household chaos/environment	1	**Other factors**	
Face mask use	7	Child's temperament	1
Duration/severity of COVID restrictions and related hardships	3	Cognitive development	4
*Educational factors*		Counting/Numeracy/Maths skills	15
ECEC/school attendance	4	Executive Functions	4
Online teaching/learning	8	Motor psychomotor development/skills	13
Learning resources	6	Non‐verbal communication	1
Relationship /communication between families and school	2	Non‐verbal intelligence	1
*Demographic factors*		Personal/Emotional/Social skills	18
Access to resources	3	Physical development	1
Caregiver's education	38	Problem‐solving	5
Caregiver's occupation	11	Reasoning	1
Children's lunch status	7	Regulation	3
Daycare/school type/location	10	Self‐monitoring	2
Household income	21	Visual discrimination	1
Household size/composition	2		
IMD or equivalent measure based on household postcode	7		
Immigration status/background	3		
Race/Ethnicity	5		
Teacher's education / experience	2		

To address objective 2, we identified factors related to COVID‐19 that affected children's home and educational environments. Within the home environment group, we identified 10 aspects, which together appeared in 61% of the corpus. Four educational factors were identified, which appeared in 21% of the studies.

To address objective 3, we scoped the data by the aspects of language development investigated. Literacy was measured in 49% of the reviewed studies, structural aspects of language such as vocabulary and syntax in 42%, and more general aspects of oral language development in 27%. Fifteen papers (16% of the corpus) investigated multilingual exposure or proficiency.

Regarding objective 4, we scoped other variables investigated alongside language in the corpus. Seventy‐four percent of papers included an analysis of non‐language variables. Personal, social, and emotional skills were investigated in 19% of studies, numeracy skills in 16%, and motor development in 14%. Other aspects of cognitive and physical development are listed in Table 4.

Regarding objective 5, we identified 11 demographic variables. Many of these were established indices of socioeconomic status (SES), e.g., caregiver education (40%) and occupation (12%), household income (22%), and indices of multiple deprivation (7%).

### Objective 6: Likely effects of factors and variables identified

Here we synthesise the evidence on the effects of the home environment, educational practices, and socioeconomic factors on language development. Some studies focused solely on how the pandemic affected factors within these categories, others looked at how aspects of the categories impacted language development, and some looked at both, i.e., the impact of the pandemic on the environment and the impact of that environment on language development.

#### Effects of the home environment

For this narrative synthesis, we separated the ten factors relating to the home environment into four categories: (a) home learning environment (comprising the home language/learning environment, home literacy practices and digital practices, and other activities happening at home); (b) other aspects of the home (parental mental health, parental practices, household chaos); (c) face mask use; and (d) severity of COVID‐19 restrictions.

Caregivers increased learning activities at home during the pandemic (Cahoon, McGill, & Simms, 2021; Izci, Geesa, Chen, & Song, 2023; Nkomo, Magxala, & Lebopa, 2023) and spent more time than before the pandemic interacting with their child, e.g., playing and helping with schoolwork/other tasks (Polat & Kesik, 2022; Schmeer, Singletary, Purtell, & Justice, 2023; Višnjić‐Jevtić & Visković, 2021). Many studies reported increased caregiver time on home literacy activities (Sonnenschein, Stites, & Ross, 2021; Wheeler & Hill, 2021), including shared reading (Gómez‐Merino, Rubio, Ávila, Gil, & Natalizi, 2023) and writing (López‐Escribano, Escudero, & Pérez‐López, 2021). Caregivers were more involved in literacy skills (Kurnia, Ramdha, & Putra, 2022), peaking in the first year of the pandemic (Li & Lin, 2023). Parental engagement was found to reduce in the later stages of the pandemic, though the availability of learning resources, e.g., books, increased over the period (Miller, Neupane, Joshi, Lohani, & Shrestha, 2023). Parents who were employed, whether outside or inside the home, spent more time reading with their children during the pandemic than before (Gómez‐Merino et al., 2023). One study reported less time spent on adult‐child reading during the pandemic compared to pre‐pandemic, though it reported an increase in digitally mediated reading (Read, Gaffney, Chen, & Imran, 2022).

Studies analysing the interaction of the home language environment and socioeconomic background found mixed results: some researchers found no association between SES and time spent on home learning activities (Cahoon et al., 2021), though caregivers in lower‐SES households were reported to hold more value in shared reading (Schmeer et al., 2023). On the other hand, children in lower‐SES families spent less time reading during the pandemic compared to their advantaged peers (Fung, St. Pierre, Raja, & Johnson, 2023) and more time watching TV or playing video games (Lampis et al., 2023). In a study with bilingual families, the pandemic was found to reduce interactions in L2 English (Li & Lin, 2023).

Significant increases in the use of digital media were found during the pandemic period (Fung et al., 2023; Gómez‐Merino et al., 2023; Sun, Tan, & Chen, 2023), especially for older children (Fung et al., 2023; Read et al., 2022). Some studies specified that this was for educational activities (Sonnenschein et al., 2021; Sonnenschein, Stites, Gursoy, & Khorsandian, 2023), including shared reading (Read et al., 2022), though when parents engaged in shared reading, Gómez‐Merino et al. (2023) found this was more likely to use conventional than digital media.

Taken together, studies commonly found a richer home language environment as a result of the pandemic, though this varied by circumstances and over time. Few studies presented outcome measures as a result of environmental changes, with three exceptions: Children who had less passive screen exposure and whose caregivers read more to them showed larger gains in vocabulary development during lockdown (Kartushina et al., 2022); more parent–child engagement led to increased communication scores (Miller et al., 2023); and children who used digital devices more scored significantly higher in reading comprehension (Lin, Molgaard, Wishard Guerra, & Cohen, 2023).

We grouped studies looking at parental mental health, parental practices, or household chaos/environment as ‘other aspects of home’. Nine of the studies we reviewed were in this category. Parenting stress was negatively associated with parents' home literacy involvement (Zambrana & Hart, 2022), but parental mental health was not found to impact vocabulary growth (McGillion et al., 2023) or wider speech and language assessment (Jeličić et al., 2021). Sensitive caregiving was positively associated with expressive vocabulary growth (McGillion et al., 2023). Although no papers directly investigated the impact of household chaos on language development, domestic conflict was positively associated with parental engagement with children's schoolwork but (as with increased household chaos) not in other learning activities (Schmeer et al., 2023).

Adult mask use was found not to impact young children's word segmentation (Frota, Pejovic, Cruz, Severino, & Vigário, 2022), word recognition (Singh & Quinn, 2023), and expressive vocabulary (Feijoo, Amadó, Sidera, Aguilar‐Mediavilla, & Serrat, 2023; Singh, Tan, & Quinn, 2021), although there were some differences in adults' perceptions of their own language quality and quantity (Crimon et al., 2022) and of children's looking behaviour (Frota et al., 2022; Singh & Quinn, 2023). Pandemic‐related hardships (Nozadi et al., 2023) related to lockdown severity or duration (Sperber, Hart, Troller‐Renfree, Watts, & Noble, 2023) were not found to directly impact language outcomes.

#### Effects of educational practices

As noted in Table 4, we identified four aspects of educational practices in the corpus, which appeared in a small proportion of studies.

Four studies investigated the effects of attendance at educational settings during the pandemic, finding positive effects on learning. Children who attended more in‐person preschool had better language and literacy skills than their peers who did not attend (Davies et al., 2021, 2023; Kilenthong, Boonsanong, Duangchaiyoosook, Jantorn, & Khruapradit, 2023; Lynch, Lee, & Loeb, 2023). Children who transitioned to in‐person attendance at school during the year performed better in reading than their peers who learned solely online or solely at school, though note that most of the children in this study were older than our age range of interest (Martinez Jr, 2022).

Remote instruction/interaction was commonly found to improve language and reading (Carney Hagan, 2022; Dore, Justice, Mills, Narui, & Welch, 2021; Richter et al., 2022), though there was variability in the performance. Children who were more engaged in remote learning showed more growth in reading (Bourassa, 2022).

Educators found online teaching challenging, e.g., being able to assess students and provide effective feedback, inequities in home resources, and modelling abstract concepts (Aslan, Li, Bonk, & Nachman, 2022; Spadafora, Reid‐Westoby, Pottruff, Wang, & Janus, 2023). Caregivers, particularly those who were employed, found online learning as a source of stress (Briesch, Codding, Hoffman, Rizzo, & Volpe, 2021; Drvodelić, Domović, & Pažur, 2021), although they were pleased with their children's learning achievements (Drvodelić et al., 2021). Good collaboration and communication between school and home facilitated learning (Serrano‐Díaz, Aragón‐Mendizábal, & Mérida‐Serrano, 2022).

Ten papers aimed to determine the efficacy of an intervention delivered during the pandemic, using a range of approaches including personalisation and technology. The majority trialled reading interventions (Baker, 2022; Bourassa, 2022; Dore et al., 2021; Klein et al., 2023; Richter et al., 2022; Silverman et al., 2023; Weiss et al., 2022), and three focused on oral language (Bennett, Gunn, Peterson, & Bellara, 2023; Khamsuk & Whanchit, 2021; Koprulu, 2021). Most reported positive effects of the intervention except for Silverman et al. (2023). Some studies claim that the parity of the intervention group's results with expected development provides evidence for effective mitigation of pandemic‐related educational disruption (Richter et al., 2022).

#### Effects of demographic factors

Sample demographics were analysed in almost half of the studies, measured using indices commonly found in the literature (see Table 3). Some of these analysed the direct role of SES in language development; others included SES as a moderating or mediating factor.

Focusing on a range of representative studies, social disadvantage was associated with reduced availability of learning resources (Sun et al., 2023); reduced reading time (Fung et al., 2023); more time on digital media (Lampis et al., 2023); reduced access to learning technology (Cahoon et al., 2021); lower use of technology for educational games (Lin et al., 2023); lower ASQ scores (Giesbrecht et al., 2023); slower growth in oral reading fluency (Domingue et al., 2022); reduced growth of component literacy skills (Borges, Koltermann, Minervino, & de Salles, 2023); the development of ‘language problems’ (Weyers & Rigó, 2023), and greater benefits of education attendance (Davies et al., 2021).

Social disadvantage was not associated with the time caregivers spent supporting their children's home‐schooling or access to learning space (Cahoon et al., 2021); the burden of home‐schooling (Drvodelić et al., 2021); and learning loss for foundational reading skills (Molnár & Hermann, 2023).

### Additional analysis of language development over time

Although not one of our planned objectives, it is useful to acknowledge the 31 studies in our corpus that used cross‐sectional or longitudinal methods to compare language outcomes in children developing during the pandemic with those developing in pre‐pandemic times, distinct from analyses of specific aspects of the environment. Most (24) evidenced a decline in language development over the pandemic period relative to trajectories from pre‐pandemic periods, though note that these are relatively short‐term comparisons.

At the earliest stages of development, deficits in social communication were found in babies born during the initial lockdown period relative to their earlier‐born peers (Byrne et al., 2023). Several studies found lower scores in communication (e.g., using the Ages and Stages Questionnaire) among cohorts growing up during the pandemic (Byrne et al., 2023; Ferrari et al., 2022; Giesbrecht et al., 2023; Nozadi et al., 2023). Others found lower levels of school readiness in pandemic‐era groups (Molnár & Hermann, 2023; Quenzer‐Alfred et al., 2021) or reduced gains in several areas of academic progress (Erbay & Tarman, 2022; Haelermans et al., 2022). In specific areas of language, scores were lower, e.g., language perception, vocabulary, and morphosyntax in pandemic‐era cohorts (Bem‐Haja, Nossa, Pereira, & Silva, 2022; Frota et al., 2022; Fung et al., 2023; Murillo, Casla, Rujas, & Lázaro, 2023; Nevo, 2023). Literacy skills (i.e., reading and writing) were commonly found to be weaker in pandemic‐era groups (Blainey & Hannay, 2021a, 2021b; Bourassa, 2022; Domingue et al., 2022; Haelermans et al., 2021; Rose et al., 2021; Schweiger, 2022; Skar, Graham, & Huebner, 2022) with children from lower‐SES backgrounds showing a greater effect. In studies with children from immigrant families, a deterioration of the language children used at school was found, relative to growth in their home or heritage language (Idoiaga Mondragon, Orcasitas‐Vicandi, & Roman Etxebarrieta, 2022; Li & Lin, 2023; Weyers & Rigó, 2023).

Seven studies showed no detriment due to the pandemic, for example, on the development of language and communication (Hadley, Liu, Kim, & McKenna, 2023; Hallin, Danielsson, Nordström, & Fälth, 2022; Imboden, Sobczak, & Griffin, 2022; Sperber et al., 2023). In three other cases, short‐term quarantine was found to be beneficial for development (Yang, Shi, Jin, & Tong, 2023), the home language grew more during the pandemic than in a pre‐pandemic group (Sheng et al., 2021), and children were found to gain more words than expected during lockdown (Kartushina et al., 2022).

## Discussion

### Summary of evidence

This scoping review collates and synthesises research investigating the effects of the COVID‐19 pandemic and its associated lockdowns on children's language environments and development. Our search generated 94 studies published in 2020–2023. These form a comprehensive evidence base documenting the unprecedented environmental changes impacting children's communicative environments at home and in educational settings. Addressing our six objectives, here we discuss the concepts studied, approaches used in the research, and themes emerging from the research findings.

#### Objective 1. To summarise the demographic and methodological characteristics of the evidence base about the impact of COVID‐19 on language development (see Table 2)

Our review reveals a diverse range of research, investigating a richness of environmental factors and domains of language development using a variety of methods and tools, from birth through the teenage years, as well as the impacts of the pandemic on parents and teachers. The majority of the evidence included in our review consisted of journal articles mainly focusing on the language development of children aged 6 years or under, using data collected during the pandemic. However, many of the papers also included a wider range of each of these aspects, reflecting the complex interaction of factors bound up in the topic. Research teams were likely motivated to collect broader datasets to increase the value of the opportunities they had during the pandemic. For example, it was not always possible to separate data of our age range of interest from that of older peers, as some papers combined a broader age range within their sample. Indeed, despite setting eligibility parameters for samples of 6 years or under, the majority of the corpus (65% of studies) also included children up to 11 years. Many papers combined data collected during COVID‐19 with data collected pre‐ or post‐pandemic, which may explain the surprisingly high proportion (27%) that used in‐person methods. Some studies conveyed the interaction of adult and child perspectives within the same study, and over half of the studies analysed language alongside other aspects of development. The most significant gap in the corpus is the lack of studies from contexts other than Europe and North America. The lack of representation of research from Asia, which accounted for 15% of the corpus, as well as from Africa, Australia, and South America, which jointly accounted for only 3% of the corpus, is particularly concerning considering the global impact of the pandemic and the fact that around 86% of the world population lives in these four continents (O'Neill, 2024). In terms of linguistic characteristics, the continents which were represented the least in this scoping review contain the highest number of threatened/endangered languages in the world (Armstrong, 2022), as well as a significant portion of the world's multilingual population, the nature of whose multilingualism (e.g., in terms of the number of languages in daily use and/or code‐switching practices) is often different from the European and Northern American contexts.

Methodologically, studies frequently relied on parental reports of language ability, using, for example, the Home Literacy Activities Questionnaire (HLAQ); Bilingual Language Background and Use Questionnaire; Communicative Development Inventories (CDI); Clinical Evaluation of Language Fundamentals (CELF); Peabody Picture Vocabulary Test (PPVT‐5); and Ages and Stages Questionnaires (ASQ‐3). Sometimes these were administered online by researchers.

Almost 90% of the studies used quantitative data. Qualitative, experiential data was relatively rare in the corpus, perhaps reflecting the limited opportunity that participants and researchers had to engage and the relative ease of using existing developmental quantitative data. Unsurprisingly, there was a bias towards data collected from the home (60%) over the educational environment (21%) due to closures during lockdown and the pressures on schools during the recovery period. There was a relative lack of intervention studies, potentially for the same reason. Studies using data from multilingual contexts were relatively scarce in the corpus, which may be related to the dominance of US‐ and UK‐based studies. The corpus is biased towards Western, educated, industrialised, rich, and democratic (WEIRD) nations in both research site and author affiliation (Henrich, Heine, & Norenzayan, 2010), highlighting the urgent need to diversify developmental research to address power imbalances and limited representations, e.g., through the use of Matharu plots (Sanderson, 2023). We would also call for further research on cross‐cultural contexts for a richer understanding of pandemic effects.

The synthesis of the study aims in Table 3 again reflects our search criteria. Priority aims were to determine language and literacy development, home literacy practices, and academic skill development. The prevalence of research on literacy development in the under‐sixes reflects models of reading development which emphasise emergent or foundational skills (Ezell & Justice, 2005). A small proportion of studies (7%) focused on adult mask‐wearing and children's use of digital media (5%), reflecting concerns about these environmental changes at the time.

#### Objective 2: To identify COVID‐19‐related factors that affected children's environments

The scoping review revealed a wide array of environmental factors rooted in the home or educational context. In 40% of papers reviewed, researchers analysed activities and practices in the home, including children's use of digital and other media (confirming that the pandemic saw a rise in the former), types of play, literacy practices, and parental engagement with these activities (which generally increased during the pandemic). The preponderance of studies focusing on the home reflects the restricted sphere of existence during the pandemic. Factors relating to caregivers themselves, e.g., parenting practices, mental health, and mask‐wearing were less well represented in the corpus (16%); these may be seen as less direct measures of a child's environment. Twenty‐one percent of papers focused on educational contexts. The provision, quality, and engagement in online learning were analysed, as well as school attendance. Together, this array of factors provides a comprehensive picture of children's environments relating to their language development during the pandemic.

#### Objective 3: To identify the factors or areas of language development investigated during the Covid‐19 pandemic

The corpus contains a wide range of language domains under study. The most common areas of interest were literacy (48% of studies), vocabulary (30%), and communication broadly defined (26%). The frequent use of parental report measures may have introduced a bias towards these domains. More specific areas of language, such as processing or morphology, were relatively rare: these abilities are typically measured under controlled experimental conditions.

The emphasis on literacy is striking: this focus reflects the importance given to early literacy and the home literacy environment (including component skills such as letter knowledge and print awareness), as well as the relative accessibility of reading by families as a powerful tool in language development. The high proportion of studies focusing on vocabulary might be influenced by the prevalence of work from UK and North American contexts, in which the focus on the word gap dominates research and policy affecting educational practices. Indeed, about half of the studies focusing on vocabulary (15/28) included data from Canada, the UK, and the US. While the word gap ideology has often been defended in the literature (e.g., Golinkoff, Hoff, Rowe, Tamis‐LeMonda, & Hirsh‐Pasek, 2019; Quigley, 2018), work such as García and Otheguy (2017), Figueroa (2024), and Cushing (2024), among others, offer a comprehensive criticism of the word gap and its colonial origins, particularly in the North American and the UK contexts.

#### Objective 4: To identify other variables investigated alongside language outcomes during COVID‐19

Almost three‐quarters of studies combined language measures with analyses of non‐language variables, including cognitive, social, and physical abilities. As well as the practical explanation acknowledged above (i.e., researchers capitalised on access to families to maximise data), the more holistic nature of the studies enables researchers to explore the association between areas of ability. It also highlights the role of language in underpinning other critical developmental skills.

#### Objective 5: To identify demographic variables moderating environmental and language factors

Sixty‐three percent of studies in the corpus collected demographic data, largely relating to SES, and spanning a range of metrics. Most of these (77%) included the SES data in their analyses. As summarised in our results, established inequalities in access to learning resources and slower developmental trajectories are reproduced in the studies. More positively, some studies did not find that SES impacted the time caregivers spent supporting children's learning, perhaps since adults across the SES were juggling competing demands. However, on balance, our review underscores the disproportionate impact of the pandemic on disadvantaged families and the widening of inequalities. The mixed findings concerning SES may stem from variability in how it was estimated across studies (e.g., income, education of caregivers, postcode, free school meals, etc.). The operationalisation of SES is an ongoing challenge (e.g. Antonoplis, 2023; O'Connell, 2019), outside of the scope of this review.

#### Objective 6: To consider the likely effects of the factors and variables identified in 2–4

Our scoping review collates language‐related environmental factors introduced by the pandemic. Its studies focus on how the pandemic impacted established influences on children's learning environments. At home, children experienced more digital media use and accrued more learning resources, engaged more in home learning, and enjoyed greater parental engagement in language and literacy‐based activities (though this varied by parental mental health). Children in bilingual households had greater exposure to the home language. Mask‐wearing enters the literature as a new environmental feature: research findings converge to show no detriment to language development within the study period. In education, engagement in remote learning benefitted language learning, as did physical attendance at school. Social disadvantage was associated with poorer home learning environments, excluding parental time. In summary, the pandemic brought advantages to some children's language‐learning environment, which may have helped to mitigate school closures. However, it exacerbated pre‐existing socioeconomic gaps.

It is less straightforward to posit evidence‐based impacts of environmental factors on children's language development within the study timeframe. Apart from a couple of studies that showed pre‐pandemic mechanisms playing out during the pandemic (i.e., parental engagement increased vocabulary Kartushina et al., 2022; Miller et al., 2023), the review did not reveal causal links. During what may be seen as a global natural experiment, it is methodologically challenging to isolate environmental factors to analyse their effects. However, our additional synthesis of cross‐sectional and longitudinal studies is informative in this regard. These studies take a more holistic approach to comparing language development in children growing up during the pandemic with those developing in pre‐pandemic times, rather than investigating specific aspects of the environment. Most evidenced a decline in language development over the pandemic period relative to trajectories from pre‐pandemic periods. Affected domains include social communication, vocabulary, morphosyntax, literacy, and language of schooling, as well as general communication skills, school readiness, and other areas of academic progress. The small number of interventions included in the review suggests positive impacts on children's language. However, we must exercise caution when interpreting their efficacy in mitigating pandemic effects. Many such studies evaluated pre‐existing interventions that had to adapt to the unforeseen pandemic, rather than setting out to address a pandemic‐related need.

### Limitations

Our eligibility criteria meant that we excluded studies focusing on the language development of neurodivergent children or those with special educational needs: a broad profile known to have suffered disproportionately during the pandemic due to challenges with new routines, homeschooling, and disrupted access to community and clinical support. Our practical restriction to papers published in English, Spanish, or Serbo‐Croatian means that important studies published in other languages may have been overlooked. During COVID‐19, a large body of rapid‐response research was made available through policy and practitioner reports, excluded from our review due to time limitations. Our decision to exclude preprints and grey literature may help safeguard quality through peer review (note that an appraisal of quality is not within the aims of a scoping review). However, publication bias may lead to an over‐reporting or over‐estimation of the impact of certain factors. Our study period (while practically necessary) restricts the review to recent papers: it is important to note that the emerging findings are relatively short‐term, and that the effects of COVID‐19 on language development are likely to develop over the next generation.

## Conclusions and recommendations

This scoping review provides an early‐stage summary of the impact of COVID‐19 on young children's environments and language development. Our synthesis of 94 studies shows that this topic is a priority concern in developmental psychology, linguistics, and education. Findings suggest that although caregivers and educational practitioners can make a significant positive impact to enrich environments, the pandemic brought about a decline in language development in multiple domains in the 4 years following the initial lockdowns.

Our synthesis will support families, practitioners, and policymakers working with pandemic‐era children as they move through education. We urge researchers, practitioners, and policymakers to collaborate in mobilising the findings to date and in setting research priorities, for example, investigating the efficacy of targeted support.

We have shown the importance of attending school and early years care for gains in language and other developmental skills. In the event of comparable future events, all means necessary should be taken to keep educational settings open. Recommendations on mask use should be led by viral risk rather than perceptions of risk to communication. Based on research findings, disadvantaged children must be prioritised in the allocation of remedial resources. Children growing up during the pandemic must be supported as they move through school to mitigate lower levels of school readiness and subsequent knock‐on effects. For professionals providing this support, as well as for researchers and funders, it will be important to track children's progress in reliable and acceptable ways over time, evaluate what works, and respond to children's needs.

For researchers, funders, and publishers, we also call for (a) pre‐registration to reduce publication bias and (2) an expansion of research focus to contexts beyond North America and Europe.

**Key points**

- To our knowledge, this is the first scoping review that synthesises peer‐reviewed literature on how the COVID‐19 pandemic impacted young children's language environments and language development.
- A wide range of language and environmental factors have been investigated through diverse methods. Most of the studies reviewed were conducted in North America and Europe.
- Taken together, the majority of studies reviewed suggest a decline in young children's language development over the pandemic period relative to trajectories from pre‐pandemic periods.
- We encourage researchers, practitioners, and policymakers to continue investigating children's language development post‐pandemic, to evaluate interventions to ensure that children impacted the most receive targeted support, and to collaborate in setting evidence‐based research priorities.

## Supporting information


**Table S1.** Search strategy used on each database.

**Appendix S1.** List of studies included in this scoping review.

## Data Availability

The completed data extraction sheet can be found on the Open Science Framework: https://osf.io/4u8dw/.
