# Supplementary material for: Annual Research Review: How did COVID‐19 affect young children's language environment and language development? A scoping review
Source: J Child Psychol Psychiatry. 2024 Dec 27;66(4):569–87. doi: 10.1111/jcpp.14102 (PMC11920612; doi:10.1111/jcpp.14102)
Supplement: Supplementary file 1 — Table S1. Search strategy used on each database. [file JCPP-66-569-s001.docx]

**Supporting Information**

**Table S1.**

*Search Strategy used on Each Database*

| **Database** | **Search Strategy** | **Search filters** |
| --- | --- | --- |
| Web of Science | #1: (((((TS=("post pandemic")) OR TS=(pandemic)) OR TS=(covid 19)) OR TS=("covid-19")) OR TS=("post-pandemic")) OR TS=(covid*)  #2: ((((((((((((((((((TS=(language )) OR TS=("language development")) OR TS=(vocabulary)) OR TS=(comprehension)) OR TS=(communication)) OR TS=(semantics)) OR TS=(phonology)) OR TS=(syntax)) OR TS=(literacy)) OR TS=(reading)) OR TS=(writing)) OR TS=(speech)) OR TS=(talk*)) OR TS=(word*)) OR TS=(understand*)) OR TS=("language acquisition")) OR TS=(produc*)) OR TS=(pragmatics)) OR TS=("expressive vocabulary")  #3: (((((((((((((((TS=(child*)) OR TS=(infant*)) OR TS=(bab*)) OR TS=(toddler*)) OR TS=("early years")) OR TS=("early childhood")) OR TS=(preschool*)) OR TS=(kindergarten*)) OR TS=(reception)) OR TS=(nurser*)) OR TS=(KS1)) OR TS=("Key Stage 1")) OR TS=(Bilingual*)) OR TS=(multilingual*)) OR TS=(caregiver*)) OR TS=(parent*)  COMBINED SEARCH: #1 AND #2 AND #3 | 2020 - 2023, Psychology or Psychology Social or Women S Studies or Pediatrics or Education Educational Research or Psychology Multidisciplinary or Psychology Developmental or Family Studies or Linguistics or Neurosciences or Language Linguistics or Psychology Educational or Behavioral Sciences or Psychology Experimental or Psychology Applied or Development Studies (Web of Science Categories) |
| OVID | #1: ("post pandemic" or pandemic or covid* or "covid 19" or "covid-19" or "post-pandemic").ab.  #2: (language or "language development" or vocabulary or comprehension or communication or semantics or phonology or syntax or literacy or reading or writing or speech or talk* or word* or understand* or "language acquisition" or produc* or pragmatics or "expressive vocabulary").ab.  #3: (Child* or infant* or bab* or toddler* or "early years" or "early childhood" or parent* or preschool* or kindergarten* or reception or nurser* or "Key Stage 1" or KS1 or bilingual* or multilingual* or caregiver*).ab.  COMBINED SEARCH: #1 AND #2 AND #3 | 2020 - 2023 |
| PubMed | ((((((("post pandemic"[Title/Abstract]) OR ("post-pandemic"[Title/Abstract])) OR ("covid 19"[Title/Abstract])) OR ("covid-19"[Title/Abstract])) OR (pandemic[Title/Abstract])) OR (covid*[Title/Abstract])) AND (((((((((((((((((child*[Title/Abstract]) OR (infant*[Title/Abstract])) OR (baby[Title/Abstract])) OR (babies[Title/Abstract])) OR (toddler*[Title/Abstract])) OR ("early years"[Title/Abstract])) OR ("early childhood"[Title/Abstract])) OR (preschool*[Title/Abstract])) OR (kindergarten*[Title/Abstract])) OR (reception[Title/Abstract])) OR (nurser*[Title/Abstract])) OR (KS1[Title/Abstract])) OR ("Key Stage 1"[Title/Abstract])) OR (bilingual*[Title/Abstract])) OR (multilingual*[Title/Abstract])) OR (caregiver*[Title/Abstract])) OR (parent*[Title/Abstract]))) AND (((((((((((((((((((language[Title/Abstract]) OR ("language development"[Title/Abstract])) OR (vocabulary[Title/Abstract])) OR (comprehension[Title/Abstract])) OR (communication[Title/Abstract])) OR (semantics[Title/Abstract])) OR (phonology[Title/Abstract])) OR (syntax[Title/Abstract])) OR (literacy[Title/Abstract])) OR (reading[Title/Abstract])) OR (writing[Title/Abstract])) OR (speech[Title/Abstract])) OR (talk*[Title/Abstract])) OR (word*[Title/Abstract])) OR (understand*[Title/Abstract])) OR ("language acquisition"[Title/Abstract])) OR (produc*[Title/Abstract])) OR (pragmatics[Title/Abstract])) OR ("expressive vocabulary"[Title/Abstract]))^^[[1]](#footnote-0)^^ | 2020 - 2023,  birth-23 months,  Newborn: birth-1 month,  Infant: 1-23 months,  Preschool Child: 2-5 years,  Child: 6-12 years |
| PsychInfo | S1: TX child* OR infant* OR bab* OR toddler* OR "early years" OR "early childhood" OR preschool* OR kindergarten* OR reception OR nurser* OR KS1 OR "Key Stage 1" OR bilingual* OR multilingual* OR caregiver* OR parent*  S2: TX language OR "language development" OR vocabulary OR comprehension OR communication OR semantics OR phonology OR syntax OR literacy OR reading OR writing OR speech OR talk* OR word* OR understand* OR "language acquisition" OR produc* OR pragmatics OR "expressive vocabulary"  S3: TX "post pandemic" OR "post-pandemic" OR "covid 19" OR "covid-19" OR covid*  COMINED SEARCH: S1 AND S2 AND S3 | 2020 - 2023,  school age (6-12 yrs) preschool age (2-5 yrs) infancy (2-23 mo) neonatal (birth-1 mo) |
| ProQuest | abstract(child* OR infant* OR bab* OR toddler* OR "early years" OR "early childhood" OR preschool* OR kindergarten OR reception OR nurser* OR KS1 OR "Key Stage 1" OR bilingual* OR multilingual* OR caregiver* OR parent*) AND abstract(language OR "language development" OR vocabulary OR comprehension OR communication OR semantics OR phonology OR syntax OR literacy OR reading OR writing OR reading OR speech OR talk* OR word* OR understand* "language acquisition" OR produc* OR pragmatics OR "expressive vocabulary") AND abstract("post-pandemic" OR "post pandemic" OR "covid-19" OR covid* OR "covid 19" OR pandemic) AND pd(20200101-20231013) | 2020 - 2023,  covid-19‎, pandemics, humans, ‎ coronaviruses, child, children,  female, pediatrics, mental health , caregivers, communication, families & family life,  parents & parenting, male, children & youth, ‎ qualitative research, foreign countries, parents,  education, cross-sectional studies, schools, child, preschool, distance education,  learning, questionnaires, infant,  school closing, mothers, ‎surveys and questionnaires,, ‎ distance learning, teachers, language,  intervention, interviews, united states, ‎ literacy, teaching, infant, newborn,  risk factors, collaboration, households,  polls & surveys, socioeconomic factors, childrens health |

1. PubMed does not allow the use of truncation on words containing 3 letters or less, hence the individual search of terms such as baby and babies, and not bab*. [↑](#footnote-ref-0)
