# Supplementary material for: Annual Research Review: How did COVID‐19 affect young children's language environment and language development? A scoping review
Source: J Child Psychol Psychiatry. 2024 Dec 27;66(4):569–87. doi: 10.1111/jcpp.14102 (PMC11920612; doi:10.1111/jcpp.14102)
Supplement: Supplementary file 2 — Appendix S1. List of studies included in this scoping review. [file JCPP-66-569-s002.docx]

# **Appendix. List of Studies Included in this Scoping Review**

Ardestani, M. M. (2023). Reading achievement based on kindergarten mode of instruction: A mixed methods study [Doctoral dissertation, Delaware Valley University]. ProQuest

Aslan, S., Li, Q., Bonk, C. J., & Nachman, L. (2022). An Overnight Educational Transformation: How did the Pandemic Turn Early Childhood Education Upside Down? *Online Learning*, *26*(2), 52-77. <https://doi.org/10.24059/olj.v26i2.2748>

Baker, A. J. (2022). *Enhancing learning in a time of crisis: A case study of a reading initiative to increase reading time among K-4 elementary school students* [Doctoral dissertation, Ashland University]. ProQuest

Bao, X., Qu, H., Zhang, R., & Hogan, T. P. (2020). Modeling Reading Ability Gain in Kindergarten Children during COVID-19 School Closures. *International Journal of Environmental Research and Public Health*, *17*(17), 6371. <https://doi.org/10.3390/ijerph17176371>

Bem-Haja, P., Nossa, P., Pereira, D. S., & Silva, C. F. (2022). Did the COVID-19 Pandemic Lockdown Harm Pre-Schoolers Learning in Portugal? Yes, but with Variations Depending on Socio-Economic Status. *Education Sciences*, *12*(10), 710. https://doi.org/10.3390/educsci12100710

Bennett, S. V., Gunn, A. M. A., Peterson, B. J., & Bellara, A. P. (2023). “Connecting to themselves and the world”: Engaging young children in read-alouds with social-emotional learning. *Journal of Early Childhood Literacy*. <https://doi.org/10.1177/14687984231196232>

Blainey, K., & Hannay, T. (2021a). The impact of school closures on autumn 2020 attainment. *RS Assessment from Hodder Education*. <https://www.risingstars-uk.com/media/Rising-Stars/Assessment/RS_Assessment_white_paper_2021_impact_of_school_closures_on_autumn_2020_attainment.pdf>

Blainey, K., & Hannay, T. (2021b). *The impact of school closures on autumn 2021 attainment*. Hodder Education. <https://www.risingstars-uk.com/getmedia/8181effc-58ef-48f7-9f78-94186578efa5/The_Impact_Of_School_Closures_May_2021>

Borges, É. P. K., Koltermann, G., Minervino, C. A. d. S. M., & de Salles, J. F. (2023). The Role of Emergent Literacy Assessment in Brazilian Portuguese Literacy Acquisition during COVID-19. *Behavioral Sciences*, *13*(6), 510. <https://doi.org/10.3390/bs13060510>

Bourassa, B. J. (2022). *Investigating the Impact of the COVID-19 School Closures and Computerized Reading Instruction in COVID and Pre-COVID Times on Elementary Student Literacy Achievement* [Doctoral dissertation, Concordia University of Wisconsin]. ProQuest

Bourke, L., Lingwood, J., Gallagher-Mitchell, T., & López-Pérez, B. (2023). The effect of face mask wearing on language processing and emotion recognition in young children. *Journal of Experimental child Psychology*, *226*, 105580. <https://doi.org/10.1016/j.jecp.2022.105580>

Briesch, A. M., Codding, R. S., Hoffman, J. A., Rizzo, C. J., & Volpe, R. J. (2021). Caregiver Perspectives on Schooling From Home During the Spring 2020 COVID-19 Closures. *School Psychology Review*, *50*(4), 546-559. <https://doi.org/10.1080/2372966X.2021.1908091>

Bulgarelli, F., & Potter, C. E. (2023). Stability and change in young children's linguistic experience during the COVID-19 pandemic: Insight from a citizen-science sample in the United States. *Multilingua*. <https://doi.org/10.1515/multi-2023-0003>

Byrne, S., Sledge, H., Franklin, R., Boland, F., Murray, D. M., & Hourihane, J. (2023). Social communication skill attainment in babies born during the COVID-19 pandemic: a birth cohort study. *Archives of Disease in Childhood*, *108*(1), 20-24. <https://doi.org/10.1136/archdischild-2021-323441>

[Byrne, S., Sledge, H., Hurley, S., Hoolahan, S., Franklin, R., Jordan, N., Boland, F., Murray, D. M., & Hourihane, J. (2023). Developmental and behavioural outcomes at 2 years in babies born during the COVID-19 pandemic: communication concerns in a pandemic birth cohort. Archives of Disease in Childhood, 108(10), 846-851. https://doi.org/10.1136/archdischild-2022-325271](https://doi.org/10.1136/archdischild-2022-325271)

Cahoon, A., McGill, S., & Simms, V. (2021). Understanding home education in the context of COVID-19 lockdown. *Irish Educational Studies*, *40*(2), 443-455. <https://doi.org/10.1080/03323315.2021.1921010>

Colbert, P.B. (2021). *A quantitative phenomenological study of the proliferated use of electronics and the impact on the communication skills of* prekindergartens [Doctoral dissertation, Gardner-Webb University]. ProQuest

Crimon, C., Barbir, M., Hagihara, H., de Araujo, E., Nozawa, S., Shinya, Y., Abboub, N., & Tsuji, S. (2022). Mask wearing in Japanese and French nursery schools: The perceived impact of masks on communication. *Frontiers in Psychology*, *13*, 5348. <https://doi.org/10.3389/fpsyg.2022.874264>

Crosson, A. C., & Silverman, R. D. (2021). Impact of COVID-19 on Early Literacy Instruction for Emergent Bilinguals. *Reading Research Quarterly*, *57*(1), 5–14. <https://doi.org/10.1002/rrq.456>

Davies, C., Hendry, A., Gibson, S. P., Gliga, T., McGillion, M., & Gonzalez-Gomez, N. (2021). Early childhood education and care (ECEC) during COVID-19 boosts growth in language and executive function. *Infant and Child Development*, *30*(4), e2241. <https://doi.org/10.1002/icd.2241>

Domingue, B. W., Dell, M., Lang, D., Silverman, R., Yeatman, J., & Hough, H. (2022). The Effect of COVID on Oral Reading Fluency During the 2020–2021 Academic Year. *AERA Open*, *8*, 233285842211202. <https://doi.org/10.1177/23328584221120254>

Dore, R., Justice, L., Mills, A. K., Narui, M., & Welch, K. (2021). Virtual Kindergarten Readiness Programming for Preschool-Aged Children: Feasibility, Social Validity, and Preliminary Impacts. *Early Education and Development*, *32*(6), 903-922. <https://doi.org/10.1080/10409289.2021.1919041>

Drvodelić, M., Domović, V., & Pažur, M. (2021). Emergency Remote Education during the COVID-19 Pandemic in Spring 2020: Parents’ Perspective: Izvanredno obrazovanje na daljinu tijekom pandemije COVID-19 u proljeće 2020: roditeljska perspektiva. *Croatian Journal Educational / Hrvatski Casopis Za Odgoj I Obrazovanje*, *23*(3), 675–707. <https://doi.org/10.15516/cje.v23i3.4511>

Erbay, F., & Tarman, I. (2022). Effect of the COVID-19 Pandemic on Language Development of Preschool Children. *Issues in Educational Research*, *32*(4), 1364–1383. <https://eric.ed.gov/?id=EJ1373164>

Feijoo, S., Amadó, A., Sidera, F., Aguilar-Mediavilla, E., & Serrat, E. (2023). Language acquisition in a post-pandemic context: the impact of measures against COVID-19 on early language development. *Frontiers in Psychology*, *14*. <https://doi.org/10.3389/fpsyg.2023.1205294>

Ferrari, E., Palandri, L., Lucaccioni, L., Talucci, G., Passini, E., Trevisani, V., & Righi, E. (2022). The Kids Are Alright (?). Infants’ Development and COVID-19 Pandemic: A Cross-Sectional Study. *International journal of public health*, *67*, 1604804. <https://doi.org/10.3389/ijph.2022.1604804>

Frota, S., Pejovic, J., Cruz, M., Severino, C., & Vigário, M. (2022). Early Word Segmentation Behind the Mask. *Frontiers in Psychology*, *13*, 879123. https://doi.org/10.3389/fpsyg.2022.879123

Fung, P., St. Pierre, T., Raja, M., & Johnson, E. K. (2023). Infants’ and toddlers’ language development during the pandemic: Socioeconomic status mattered. *Journal of Experimental Child Psychology*, *236*, 105744. <https://doi.org/10.1016/j.jecp.2023.105744>

García González, E., Liu, L., & Lanza, E. (2023). Language in multilingual families during the COVID-19 pandemic in Norway: A survey of challenges and opportunities. *Multilingua*. <https://doi.org/10.1515/multi-2023-0011>

Giesbrecht, G. F., Lebel, C., Dennis, C.-L., Silang, K., Xie, E. B., Tough, S., McDonald, S., & Tomfohr-Madsen, L. (2023). Risk for Developmental Delay Among Infants Born During the COVID-19 Pandemic. *Journal of developmental and behavioral pediatrics*, *44*(6), E412-E420. <https://doi.org/10.1097/DBP.0000000000001197>

Gómez-Merino, N., Rubio, A., Ávila, V., Gil, L., & Natalizi, F. (2023). Efectos del teletrabajo y la digitalización en la lectura compartida entre padres e hijos. *Bordón. Revista de Pedagogía*, *75*(1), 65–81. <https://doi.org/10.13042/Bordon.2023.94648>

Hadley, E. B., Liu, S., Kim, E. S., & McKenna, M. (2023). State-Funded Pre-K and Children’s Language and Literacy Development: The Case of COVID-19. *Educational Researcher*, *52*(7), 434 - 443. <https://doi.org/10.3102/0013189x231179111>

[Haelermans, C., Jacobs, M., van Vugt, L., Aarts, B., Abbink, H., Smeets, C., van der Velden, R., & van Wetten, S. (2021). A full year COVID-19 crisis with interrupted learning and two school closures: The effects on learning growth and inequality in primary education. Available at https://doi.org/10.31219/osf.io/78fje](https://doi.org/10.31219/osf.io/78fje)

Haelermans, C., Korthals, R., Jacobs, M., de Leeuw, S., Vermeulen, S., van Vugt, L., Aarts, B., Prokic-Breuer, T., van der Velden, R., van Wetten, S., & de Wolf, I. (2022). Sharp increase in inequality in education in times of the COVID-19-pandemic. *PLOS ONE*, *17*(2), e0261114. <https://doi.org/10.1371/journal.pone.0261114>

Hagan, M. C. (2022). *Constructivist grounded theory approach to understanding teaching kindergarteners how to read during the COVID-19* [Doctoral dissertation, St John’s University]. ProQuest

Hallin, A. E., Danielsson, H., Nordström, T., & Fälth, L. (2022). No learning loss in Sweden during the pandemic evidence from primary school reading assessments. *International Journal of Educational Research*, *114*, 102011. <https://doi.org/10.1016/j.ijer.2022.102011>

Hatoss, A. (2023). Shifting ecologies of family language planning: Hungarian Australian families during COVID-19. *Current Issues in Language Planning*, 1–21. <https://doi.org/10.1080/14664208.2023.2205793>

Idoiaga Mondragon, N., Orcasitas-Vicandi, M., & Roman Etxebarrieta, G. (2022). Impact of emergency eLearning in a multilingual context with a minority language: how has the absence of school affected the use of Basque, English, and Spanish in the Basque context? *International Journal of Bilingual Education and Bilingualism*, *25*(10), 3533-3550. <https://doi.org/10.1080/13670050.2022.2065877>

Imboden, A., Sobczak, B. K., & Griffin, V. (2022). The impact of the COVID-19 pandemic on infant and toddler development. *Journal of the American Association of Nurse Practitioners*, *34*(3), 509-519. <https://doi.org/10.1097/JXX.0000000000000653>

Izci, B., Geesa, R. L., Chen, S., & Song, H. S. (2023). Home Learning Environments During the COVID-19 Pandemic: Caregivers' and Children's Perceptions. *Journal of Research in Childhood Education*, *37*(4), 493-505. <https://doi.org/10.1080/02568543.2022.2143459>

Jeličić, L., Sovilj, M., Bogavac, I., Drobnjak, A. e., Gouni, O., Kazmierczak, M., & Subotić, M. (2021). The Impact of Maternal Anxiety on Early Child Development During the COVID-19 Pandemic. *Frontiers in psychology*, *12*, 792053. <https://doi.org/10.3389/fpsyg.2021.792053>

Kartushina, N., Mani, N., Aktan-Erciyes, A., Alaslani, K., Aldrich, N. J., Almohammadi, A., Alroqi, H., Anderson, L. M., Andonova, E., Aussems, S., Babineau, M., Barokva, M., Bergmann, C., Cashon, C., Custode, S., de Carvalho, A., Domotrova, N., Dynak, A., Farah, R., ... & Mayor, J. (2022). COVID-19 first lockdown as a window into language acquisition: associations between caregiver-child activities and vocabulary gains. *Language Development Research*, *2*(1). [https://doi.org10.34842/abym-xv34](about:blank)

Khamsuk, A., & Whanchit, W. (2021). Storytelling: An Alternative Home Delivery of English Vocabulary for Preschoolers during COVID-19's Lockdown in Southern Thailand. *South African Journal of Childhood Education*, *11*(1), 1-13. <https://doi.org/10.4102/SAJCE.V11I1.897>

Kilenthong, W. T., Boonsanong, K., Duangchaiyoosook, S., Jantorn, W., & Khruapradit, V. (2023). Learning losses from school closure due to the COVID-19 pandemic for Thai kindergartners. *Economics of Education Review*, *96*, 102455. <https://doi.org/10.1016/j.econedurev.2023.102455>

Klein, P. D., Casola, M., Dombroski, J. D., Giese, C., Sha, K. W.-Y., & Thompson, S. C. (2023). Response to Intervention in Virtual Classrooms with Beginning Writers. *Reading & Writing Quarterly*, *39*(5), 413-435. <https://doi.org/10.1080/10573569.2022.2131662>

Köprülü, F. (2021). The Effect of Using Technology Supported Material in Teaching English to First-Year Primary School Children: On Their Academic Success During COVID-19. *Frontiers in Psychology*, *12*, 756295. <https://doi.org/10.3389/fpsyg.2021.756295>

Kurnia, R., Ramdha, T., & Putra, Z. H. (2022). Implementation of Early Literacy Activities during COVID-19: A Parents Involvement Analysis. *International Journal of Instruction*, *15*(2), 831 – 846 <https://doi.org/10.29333/iji.2022.15245a>

Kurupınar, A., & Kanmaz, T. (2023). A Study into Learning Losses of Preschool Children in Covid-19 Pandemic. *Eğitimde Nitel Araştırmalar Dergisi*, (35), 24-55. <https://doi.org/10.14689/enad.35.1569>

Kyuchukov, H. (2022). Language use of Russian Roma children in their home environments during the COVID 19 pandemic. *Intercultural Education*, *33*(1), 114-119. <https://doi.org/10.1080/14675986.2021.1966912>

Lampis, V., Mascheretti, S., Cantiani, C., Riva, V., Lorusso, M. L., Lecce, S., Molteni, M., Antonietti, A., & Giorgetti, M. (2023). Long-Lasting Effects of Changes in Daily Routine during the Pandemic-Related Lockdown on Preschoolers’ Language and Emotional–Behavioral Development: A Moderation Analysis. *Children*, *10*(4), 656. <https://doi.org/10.3390/children10040656>

Li, G., & Lin, Z. (2023). In and Out of the Unknown: Lessons from Immigrant Families Promoting Multiliteracies During the COVID‐19 Pandemic. *The Reading Teacher*, *76*(5), 570-577. <https://doi.org/10.1002/trtr.2184>

Li, G., Zhen, F., Lin, Z., & Gunderson, L. (2023). Bilingual Home Literacy Experiences and Early Biliteracy Development among Chinese–Canadian First Graders. *Education Sciences*, *13*(8), 808. <https://doi.org/10.3390/educsci13080808>

Lin, N. T., Molgaard, M., Wishard Guerra, A., & Cohen, S. (2023). Young children and families’ home literacy and technology practices before and during COVID-19. *Journal of Early Childhood Research*, *21*(3), 341-354. <https://doi.org/10.1177/1476718X231164132>

López-Escribano, C., Escudero, A., & Pérez-López, R. (2021). An Exploratory Study about Patterns of Parental Home Literacy Activities during the COVID-19 Confinement among Spanish Families. *Early education and development*, *32*(6), 812-829. <https://doi.org/10.1080/10409289.2021.1916184>

Lynch, K., Lee, M., & Loeb, S. (2023). An investigation of Head Start preschool children's executive function, early literacy, and numeracy learning in the midst of the COVID-19 pandemic. *Early Childhood Research quarterly*, *64*, 255-265. <https://doi.org/10.1016/j.ecresq.2023.04.002>

Martinez Jr, I. (2022).  *Navigating through a pandemic (the unknown): The effects of synchronous learning for online and in person students on achievement scores in reading and math in a private catholic school in South Texas* [Doctoral dissertation, The University of Texas Rio Grande Valley]. ProQuest

McGillion, M., Davies, C., Kong, S. P., Hendry, A., & Gonzalez-Gomez, N. (2023). Caregiver sensitivity supported young children's vocabulary development during the Covid-19 UK lockdowns. *Journal of Child Language*, *20*(2), 1-17. <https://doi.org/10.1017/S0305000923000211>

Miller, L. C., Neupane, S., Joshi, N., Lohani, M., & Shrestha, B. (2023). Trajectories of child growth, child development, and home child‐rearing quality during the Covid pandemic in rural Nepal. *Child: care, health & development*, *49*(5), 800-810. <https://doi.org/10.1111/cch.13078>

Molnár, G., & Hermann, Z. (2023). Short- and long-term effects of COVID-related kindergarten and school closures on first- to eighth-grade students’ school readiness skills and mathematics, reading and science learning. *Learning and Instruction*, *83*, 101706. <https://doi.org/10.1016/j.learninstruc.2022.101706>

Murillo, E., Casla, M., Rujas, I., & Lázaro, M. (2023). El efecto de la pandemia sobre el desarrollo del lenguaje en los dos primeros años de vida. *Revista de Logopedia, Foniatría Y Audiología*, *43*(3), 100315. <https://doi.org/10.1016/j.rlfa.2023.100315>

Nevo, E. (2023). The Effect of the COVID-19 Pandemic on Low SES Kindergarteners' Language Abilities. *Early Childhood Education Journal*, 1-11. <https://doi.org/10.1007/s10643-023-01444-4>

Nkomo, S. A., Magxala, X. P., & Lebopa, N. (2023). Early literacy experiences of two children during Covid-19 lockdown in South Africa: A semi- ethnographic study. *Journal of Early Childhood Literacy*, *23*(1), 141-174. <https://doi.org/10.1177/14687984231154351>

Nozadi, S. S., Li, X., Kong, X., Rennie, B., Kanda, D., MacKenzie, D., Luo, L., Posner, J., Blackwell, C. K., Croen, L. A., Ferrara, A., O’Connor, T. G., Zimmerman, E., Ghassabian, A., Leve, L. D., Elliott, A. J., Schmidt, R. J., Sprowles, J. L. N., & Lewis, J. L. (2023). Effects of COVID-19 Financial and Social Hardships on Infants’ and Toddlers’ Development in the ECHO Program. *International Journal of Environmental Research and Public Health*, *20*(2), 1013. <https://doi.org/10.3390/ijerph20021013>

Nugraha, D. S., Rafly, Z., Boeriswati, E., & Hasanah, E. (2023). Parent-Preschooler Kid’s Activities in the time of COVID-19 Outbreak: an Autoethnography on Child’s Second Language Acquisition. *Psychological Science and Education*, *28*(1), 122–131. <https://doi.org/10.17759/pse.2023280107>

Polat, İ. & Kesik, C. (2022), Parents' Views on Initial Literacy Teaching in the Distance Education Process, *Çukurova University. Faculty of Education Journal*, *51*(1), 443-472. <https://doi.org/10.14812/cuefd.947383>

Quenzer-Alfred, C., Schneider, L., Soyka, V., Harbrecht, M., Blume, V., & Mays, D. (2021). No nursery 'til school - the transition to primary school without institutional transition support due to the COVID-19 shutdown in Germany. *European Journal of Special Needs Education*, *36*(1), 127-141. <https://doi.org/10.1080/08856257.2021.1872850>

Read, K., Gaffney, G., Chen, A., & Imran, A. (2022). The Impact of COVID-19 on Families’ Home Literacy Practices with Young Children. *Early Childhood Education Journal*, *50*(8), 1429-1438. <https://doi.org/10.1007/s10643-021-01270-6>

[Richter, C. G., Siegelman, N., Mahaffy, K., Van Den Bunt, M., Kearns, D. M., Landi, N., Sabatini, J., Pugh, K., & Hoeft, F. (2022). The impact of computer–assisted technology on literacy acquisition during COVID-19-related school closures: Group–level effects and predictors of individual–level outcomes. Frontiers in Psychology, 13, 1001555. https://doi.org/10.3389/fpsyg.2022.1001555](https://doi.org/10.3389/fpsyg.2022.1001555)

Rose, S., Badr, K., Fletcher, L., Paxman, T., Lord, P., Rutt, S., Styles, B., & Twist, L. (2021). Impact of School Closures and Subsequent Support Strategies on Attainment and Socio-Emotional Wellbeing in Key Stage 1. Research Report. In *ERIC*. Education Endowment Foundation. <https://eric.ed.gov/?id=ED620409>

Schmeer, K. K., Singletary, B., Purtell, K. M., & Justice, L. M. (2023). Family Disruption and Parenting During the COVID-19 Pandemic. *Journal of family issues*, *44*(1), 112-138. <https://doi.org/10.1177/0192513X211042852>

Schweiger, E. M. (2022). *Literacy and COVID-19: Elementary students’ reading performance through a global pandemic* [Doctoral dissertation, State University of New York]. ProQuest

Seo, S., & Song, J. (2023). Toddler–teacher interaction and teachers’ sensitivity as predictors of toddler’s development during COVID-19: Stability or change over time. *Frontiers in Psychology*, *14*, 1161947. <https://doi.org/10.3389/fpsyg.2023.1161947>

Serrano-Díaz, N., Aragón-Mendizábal, E., & Mérida-Serrano, R. (2022). Families' perception of children's academic performance during the COVID-19 lockdown. *Comunicar*, *30*(70), 53-62. <https://doi.org/10.3916/C70-2022-05>

Sheng, L., Wang, D., Walsh, C., Heisler, L., Li, X., & Su, P. L. (2021). The Bilingual Home Language Boost Through the Lens of the COVID-19 Pandemic. *Frontiers in Psychology*, *12*, 667836. <https://doi.org/10.3389/fpsyg.2021.667836>

Silverman, R. D., Keane, K., Hsieh, H., Southerton, E., Scott, R. C., & Brunskill, E. (2023). Texting and tutoring: Short-term K-3 reading interventions during the pandemic. *The Journal of Educational Research, 116*(5), 254-267. <https://doi.org/10.1080/00220671.2023.2251432>

Singh, L., & Quinn, P. C. (2023). Effects of face masks on language comprehension in bilingual children. *Infancy*, *28*(4), 738-753. <https://doi.org/10.1111/infa.12543>

Singh, L., Tan, A., & Quinn, P. C. (2021). Infants recognize words spoken through opaque masks but not through clear masks. *Developmental science*, *24*(6), e13117. <https://doi.org/10.1111/desc.13117>

Skar, G. B. U., Graham, S., & Huebner, A. (2022). Learning Loss During the COVID-19 Pandemic and the Impact of Emergency Remote Instruction on First Grade Students’ Writing: A Natural Experiment. *Journal of educational psychology*, *114*(7), 1553-1566. <https://doi.org/10.1037/edu0000701>

Sonnenschein, S., Stites, M. L., Gursoy, H., & Khorsandian, J. (2023). Elementary-School Students’ Use of Digital Devices at Home to Support Learning Pre- and Post-COVID-19. *Education sciences*, *13*(2), 117. <https://doi.org/10.3390/educsci13020117>

Sonnenschein, S., Stites, M., & Ross, A. (2021). Home learning environments for young children in the US during COVID-19. *Early Education and Development*, *32*(6), 794-811. <https://doi.org/10.1080/10409289.2021.1943282>

Spadafora, N., Reid-Westoby, C., Pottruff, M., Wang, J., & Janus, M. (2023). From Full Day Learning to 30 Minutes a Day: A Descriptive Study of Early Learning During the First COVID-19 Pandemic School Shutdown in Ontario. *Early Childhood Education Journal*, *51*(2), 287-299. <https://doi.org/10.1007/s10643-021-01304-z>

Sperber, J. F., Hart, E. R., Troller‐Renfree, S. V., Watts, T. W., & Noble, K. G. (2023). The effect of the COVID‐19 pandemic on infant development and maternal mental health in the first 2 years of life. *Infancy*, *28*(1), 107-135. <https://doi.org/10.1111/infa.12511>

Steinmayr, R., Lazarides, R., Wirthwein, L., & Christiansen, H. (2023). Comparison of Parent-Rated Teaching Activities during the First and Second School Lockdowns and Its Association with Students' Learning Outcomes during Distant Teaching. *Zeitschrift für Psychologie*, *231*(3), 192-203. <https://doi.org/10.1027/2151-2604/a000528>

Stucke, N. J., Stoet, G., & Doebel, S. (2022). What are the kids doing? Exploring young children's activities at home and relations with externally cued executive function and child temperament. *Developmental Science*, *25*(5), e13226. <https://doi.org/10.1111/desc.13226>

Sun, H., Tan, J., & Chen, W. (2023). COVID-19 and bilingual children’s home language environment: Digital media, socioeconomic status, and language status. *Frontiers in Psychology*, *14*, 1115108. <https://doi.org/10.3389/fpsyg.2023.1115108>

Sun, X., Marks, R. A., Eggleston, R. L., Zhang, K., Lau, C., Yu, C.-L., Nickerson, N., & Kovelman, I. (2023). Impacts of the COVID-19 disruption on the language and literacy development of monolingual and heritage bilingual children in the United States. *Reading and Writing*, *36*(2), 347-375. <https://doi.org/10.1007/s11145-022-10388-x>

Surrain, S., Mesa, M. P., Assel, M. A., & Zucker, T. A. (2023). Does Assessor Masking Affect Kindergartners’ Performance on Oral Language Measures? A COVID-19 Era Experiment With Children From Diverse Home Language Backgrounds. *Language, Speech & Hearing Services in Schools*, *54*(4), 1323-1332. <https://doi.org/10.1044/2023_LSHSS-22-00197>

Višnjić-Jevtić, A., & Visković, I. (2021). Roditeljstvo u vrijeme pandemije Covid-19: perspektiva roditelja djece rane i predškolske dobi. *Metodički ogledi: časopis za filozofiju odgoja*, *28*(1), 11-38. <https://doi.org/10.21464/mo.28.1.4>

Weiss, Y., Yeatman, J. D., Ender, S., Gijbels, L., Loop, H., Mizrahi, J. C., Woo, B. Y., & Kuhl, P. K. (2022). Can an Online Reading Camp Teach 5-Year-Old Children to Read? *Frontiers in Human Neuroscience*, *16*, 793213. <https://doi.org/10.3389/fnhum.2022.793213>

Weyers, S., & Rigó, M. (2023). Child health and development in the course of the COVID-19 pandemic: are there social inequalities? *European Journal of Pediatrics*, *182*(3), 1173-1181. <https://doi.org/10.1007/s00431-022-04799-9>

Wheeler, D. L., & Hill, J. C. (2021). The impact of COVID-19 on early childhood reading practices. *Journal of early childhood literacy*, *0*(0). https://doi.org/10.1177/14687984211044187

Yang, T., & Zhang, Y. (2023). An Ecological Investigation of Kindergarten-Oriented Educational Practice during the Initial COVID-19 Class Suspension in China. *Early Education and Development*, *34*(5), 1191-1212. <https://doi.org/10.1080/10409289.2022.2095858>

Yang, Y., Shi, L., Jin, X., & Tong, S. (2023). Effects of short-term quarantine on growth and development of children aged 1–36 months during the Omicron outbreak. *European Journal of pediatrics*, *182*(3), 1351-1359. <https://doi.org/10.1007/s00431-023-04817-4>

Zambrana, K. A., & Hart, K. C. (2022). Riesgo Y Resiliencia: Exploring the Role of Parenting Stress and Self-efficacy on Young Latino Children's Well-being and Home Learning Experiences during COVID-19. *Journal of Latinos and Education*, *21*(3), 212-223. <https://doi.org/10.1080/15348431.2022.2051037>
